# A CAD system for automatic dysplasia grading on H&E cervical whole-slide images

**DOI:** 10.1038/s41598-023-30497-z

**Published:** 2023-03-09

**Authors:** Sara P. Oliveira, Diana Montezuma, Ana Moreira, Domingos Oliveira, Pedro C. Neto, Ana Monteiro, João Monteiro, Liliana Ribeiro, Sofia Gonçalves, Isabel M. Pinto, Jaime S. Cardoso

**Affiliations:** 1 INESCTEC, 4200-465 Porto, Portugal; 2 FEUP, University of Porto, 4200-465 Porto, Portugal; 3 IMP Diagnostics, 4150-146 Porto, Portugal; 4 ICBAS, University of Porto, 4050-313 Porto, Portugal

**Keywords:** Cervical cancer, Image processing, Machine learning, Pathology

## Abstract

Cervical cancer is the fourth most common female cancer worldwide and the fourth leading cause of cancer-related death in women. Nonetheless, it is also among the most successfully preventable and treatable types of cancer, provided it is early identified and properly managed. As such, the detection of pre-cancerous lesions is crucial. These lesions are detected in the squamous epithelium of the uterine cervix and are graded as low- or high-grade intraepithelial squamous lesions, known as LSIL and HSIL, respectively. Due to their complex nature, this classification can become very subjective. Therefore, the development of machine learning models, particularly directly on whole-slide images (WSI), can assist pathologists in this task. In this work, we propose a weakly-supervised methodology for grading cervical dysplasia, using different levels of training supervision, in an effort to gather a bigger dataset without the need of having all samples fully annotated. The framework comprises an epithelium segmentation step followed by a dysplasia classifier (non-neoplastic, LSIL, HSIL), making the slide assessment completely automatic, without the need for manual identification of epithelial areas. The proposed classification approach achieved a balanced accuracy of 71.07% and sensitivity of 72.18%, at the slide-level testing on 600 independent samples, which are publicly available upon reasonable request.

## Introduction

Cervix uteri cancer is the fourth most prevalent cancer, the fourth cause of cancer-related death in women, worldwide, and the ninth leading cause of female cancer, in Europe^1^. Around 80–90% of cervical cancers are squamous cell carcinomas (SCC) and the large majority of these are caused by human papillomavirus (HPV) infection, although HPV-independent forms of cancer also exist^2^. Fortunately, cervical cancer is one of the most successfully preventable and treatable forms of cancer, as long as it is detected early and effectively managed^3^. Thus, screening pre-cancerous lesions and vaccination are key to preventing the disease. Squamous intraepithelial lesions (SILs) of the uterine cervix, or cervical intraepithelial neoplasia (CIN), are pre-malignant HPV-driven proliferations of the squamous epithelium, showing viral cytopathic changes and/or maturation alterations that do not extend beyond the basement membrane^2^. Importantly, the World Health Assembly adopted a global strategy for cervical cancer elimination in August 2020, which is set on 3 pillars: vaccination, screening and treatment^4,5^. Each country should meet the 90–70–90 targets, by 2030: 90% of girls fully vaccinated against HPV by the age of 15; 70% of women screened using a high-performance test by the age of 35, and again by the age of 45; 90% of women with pre-cancer treated and 90% of women with invasive cancer managed^4,5^. Thus, technological innovation (new and better tools) is needed to achieve cervical cancer screening and management targets, as stated by Rodriguez et al.^6^, as well as the strategic implementation of automatic diagnosis tools in clinical practice. In computational pathology, there are several examples of studies focused on invasive cancer detection^7–9^ and grading^10–12^, but in cervical pathology, detecting and grading intraepithelial neoplasia has paramount significance, as detection of the disease in a precancerous state allows for close monitoring and effective treatment, preventing the development of cancer. To this end, the main contributions of this work are (1) a computer-aided diagnosis (CAD) system for dysplasia grading on cervical whole-slide images (WSI), which includes (2) a segmentation model that firstly identifies squamous epithelium tissue and (3) a weakly-supervised classifier to diagnose dysplasia from digitised Haematoxylin–Eosin (H &E) stained cervical slides. Moreover, (4) the data set used for testing, with 600 slides from LEEP samples and surgical specimens, from the IMP Diagnostics archive, is publicly available.

## Cervical cancer pathology

The grading of cervical dysplasia is currently based on a two-tier system (LSIL/HSIL), as it shows enhanced reproducibility between pathologists and higher biological significance when compared with the previously used system (CIN 1/2/3)^2^. Grading is mostly based on the proportion and location of immature cells within the squamous epithelium and on the cytopathic changes caused by HPV.

**Figure 1:**
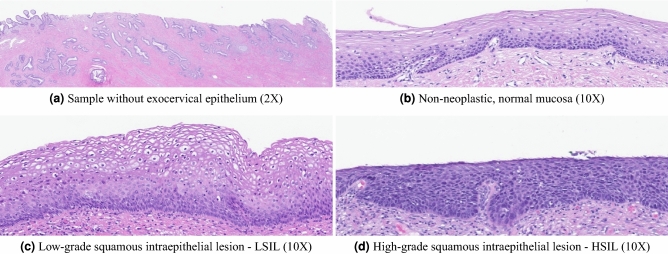
Non-representative sample (**a**); cervical squamous epithelium dysplastic progression (**b**,**c**).

According to the WHO classification of tumours, low-grade squamous intraepithelial lesions (LSIL) are characterised by the proliferation of basal/parabasal-like (immature looking) cells within the lower third of the epithelium (Fig. 1c), along with the so-called koilocytic atypia (i.e. recognisable nuclear and cytoplasmic changes caused by HPV, namely characteristic perinuclear vacuolisation and nuclear enlargement, irregularity and hyperchromasia)^2^. Mitotic activity can be observed, but atypical mitoses should be absent or rare. In contrast, in high-grade squamous intraepithelial lesions (HSIL), the proliferation of basal/parabasal-like cells extends to the middle and upper thirds of the epithelium (Fig. 1d). Nuclear abnormalities are seen throughout the thickness of the epithelium as well as mitotic activity (including atypical mitosis)^2^. The term HSIL encompasses the formerly known CIN2 and CIN3 lesions, as well as carcinoma in situ lesions. It is known that, similarly to other grading tasks in pathology, grading cervical dysplasia is hurdled by significant inter- and intra-observer variability^13^. Nonetheless, distinguishing normal mucosa, LSIL and HSIL has major clinical implications and remains an important task in gynaecological pathology. The main interpretative problems in this context are the distinction of normal mucosa (Fig. 1b), which can show nonspecific inflammatory/reactive changes, from dysplasia (LSIL) and also to distinguish benign, mostly transient, dysplasia (LSIL) from pre-cancer (HSIL)^14^. Using ancillary markers, namely p16 staining, can help in some cases, but its overuse should be avoided, since it might cause over-diagnosis of high-grade lesions, and it should not exceed 20–40% (or even less) of cervical biopsies^2^.

## Related work

Computational pathology studies applied to cervical cancer have been mostly focused on cytology^15–18^, since the initial screening of lesions is performed using cytology specimens (smears or liquid-based preparations), either by HPV molecular testing or co-testing HPV in combination with cytology evaluation^19–21^. Nonetheless, it is the histologic assessment of cervical biopsies, loop electrosurgical excision procedure (LEEP) samples and surgical specimens that constitutes the gold standard for the diagnosis of cervical lesions. As such, cervical histopathology image analysis is also an active research field^22^. Noteworthy, in the last 5 years, most classification studies using deep-learning approaches in cervical cancer have only used cropped images as opposed to full WSIs.

In 2019, Li et al.^23^, proposed a transfer learning framework of Inception-V3 network to classify well, moderately and poorly differentiated cervical carcinomas. They used 307 IHC-stained images (with AQP, HIF and VEGF immunomarkers) and reported a 77.3% classification accuracy. Further, also in 2019, the authors presented an improved method for the same task, with 88% global accuracy^24^. In this study, a weakly supervised framework, based on multilayer-hidden conditional random fields, was developed on a dataset of more than 100 IHC-stained images. Also in 2019, a study by Xue et al.^25^ analysed the 4-class (normal, CIN 1 to 3) classification problem, using conditional Generative Adversarial Networks (cGANs) to expand the training dataset, by synthesising realistic cervical images. They report using 1112 normal, 181 CIN1, 463 CIN2 and 454 CIN3 patches of $256\times 128$ pixels, although not specifying from how many patients/samples the patches derive. Their results show a significant improvement in classification accuracy from 66.3 to 71.7%, using the same ResNet-18 baseline classifier, after leveraging with the cGAN-generated images. In 2021, the same group also proposed a synthetic augmentation framework that selectively adds new synthetic image patches, generated by their GAN model (HistoGAN), rather than expanding directly the training set with synthetic images^26^. This experiment, using a similar cervical dataset, and an additional dataset of metastasised lymph node images, resulted in significant and consistent improvement of the classification performance: 6.7% and 2.8% higher accuracy, compared with their previous work, for cervical histopathology and metastatic cancer datasets, respectively. In 2020, Huang et al.^27^ proposed a classification method based on the least absolute shrinkage and selection operator (LASSO) and ensemble learning support vector machine (EL-SVM). Images from 468 cervical biopsies were used, and an 86.84% average accuracy was shown, classifying normal, LSIL and HSIL lesions and carcinoma. Alternately, Sornapudi et al.^28^ have built a network pipeline (DeepCIN), containing two classifier networks, to analyse high-resolution images ($n=453$, manually extracted from 146 WSIs), which achieved an 88.5% accuracy (normal, CIN 1 to 3). In 2021, Huang et al.^29^ proposed the AF-SENet, Analysis of Variance-F value-Spectral Embedding Net, to classify cervical lesions by fusing features extracted with the ResNet50v2 and DenseNet121 networks with an (ANOVA F)-spectral embedding strategy. The authors have used a total of 468 images, with a resolution of $3456\times 4608$, with 150 being normal, 85 LSIL, 104 HSIL and 129 carcinoma and reported an average classification accuracy of 95.33%. In the same year, Albayrak et al.^30^ reported a classification accuracy of 65.4% similarly grading cervical precursor lesions (normal, CIN 1 to 3), using a morphological-based feature extraction method. Their dataset consisted of 128 images from 54 patients. In 2022, Cho et al.^31^, aimed to develop and validate deep learning (DL) models to classify cervical intraepithelial neoplasia (normal, CIN1 to 3) automatically. The models were developed on a dataset comprising 1106 images from 588 patients, and the mean accuracies for the four-class classification were 88.4% by DenseNet-161 and 89.5% by EfficientNet-B7, which were similar to the reported performance of two pathologists (93.2% and 89.7%). Further, they also calculated the performance for a three-class classification (correspondent to normal, LSIL, and joining CIN2 and CIN3 as HSIL), and the mean accuracies of DenseNet-161 and EfficientNet-B7 increased to 91.4% and 92.6%, respectively (whereas pathologists’ performances were 95.7% and 92.3%). Lastly, Habtemariam et al.^32^ have also proposed a cervical cancer classification system using DL techniques. In this study, the authors resorted to 915 histopathology images (and also included 4005 colposcopy images) and reported a test accuracy of 94.5% for cervical cancer classification into normal, pre-cancer, squamous cell carcinoma and adenocarcinoma), using the Efficientnet-B0 model. Regarding the colposcopy images, the model achieved an accuracy of 96.84% for cervix-type classification.

Working on such big images as WSI has the increased difficulty of high dimensionality and resolution, which can not be easily fitted in graphics process units (GPU), usually used to train DL models. Nevertheless, it is the most significant, as it is the only way for models to be effectively translated into clinical practice. Therefore, despite the promising results, the fact that the approaches presented before, firstly, need a manual selection of the areas to be classified and, secondly, only focus on small regions (considering the size of the slide), makes them more fragile from an application point of view. With an automatic segmentation step, there is no need for annotation or manual cropping when analysing a new sample. On the other hand, a slide-wise classification takes into account all the information available within the WSI, which can be highly heterogeneous. Moreover, even if the tissue classifier performs well on smaller regions (tiles or crops), the segmentation and aggregation steps have their own challenges and can impact a slide assessment. Thus, direct comparisons should be drawn carefully, and it is not clear how such models would perform in a full pipeline.

To the best of our knowledge, regarding classification tasks on cervical pathology, only the work of Sornapudi et al.^33^, in 2021, was developed directly on WSI. They have developed a novel image analysis toolbox to automate CIN diagnosis of cervical biopsies, with an 85% exact-class accuracy. This work shows the potential of the used methodology, but it was trained with only 150 WSI, which raises concerns about generalisation performance with larger and more heterogeneous data sets. Thus, in an attempt to improve on these limitations, we propose a new approach developed with a set of 2000 cervical slides, collected from the IMP Diagnostics archive.

## Material and methods

### Problem definition

As mentioned earlier, automatic cervical dysplasia grading should be done on H &E WSI and focused on the cells of the squamous epithelium, that on LEEP samples and surgical specimens is usually a thin area within the sample tissue. Thus, despite the usual big dimension of digitised slides, which easily become hard and tedious to annotate^34^, fully-supervised models for cervical dysplasia may require annotation and labelling of epithelium, besides the usual annotation of relevant areas for diagnosis/classification. However, in general, WSI are not usually publicly available, and, when they are, they have only the associated diagnosis and no detailed pixel-level annotations.

**Figure 2:**
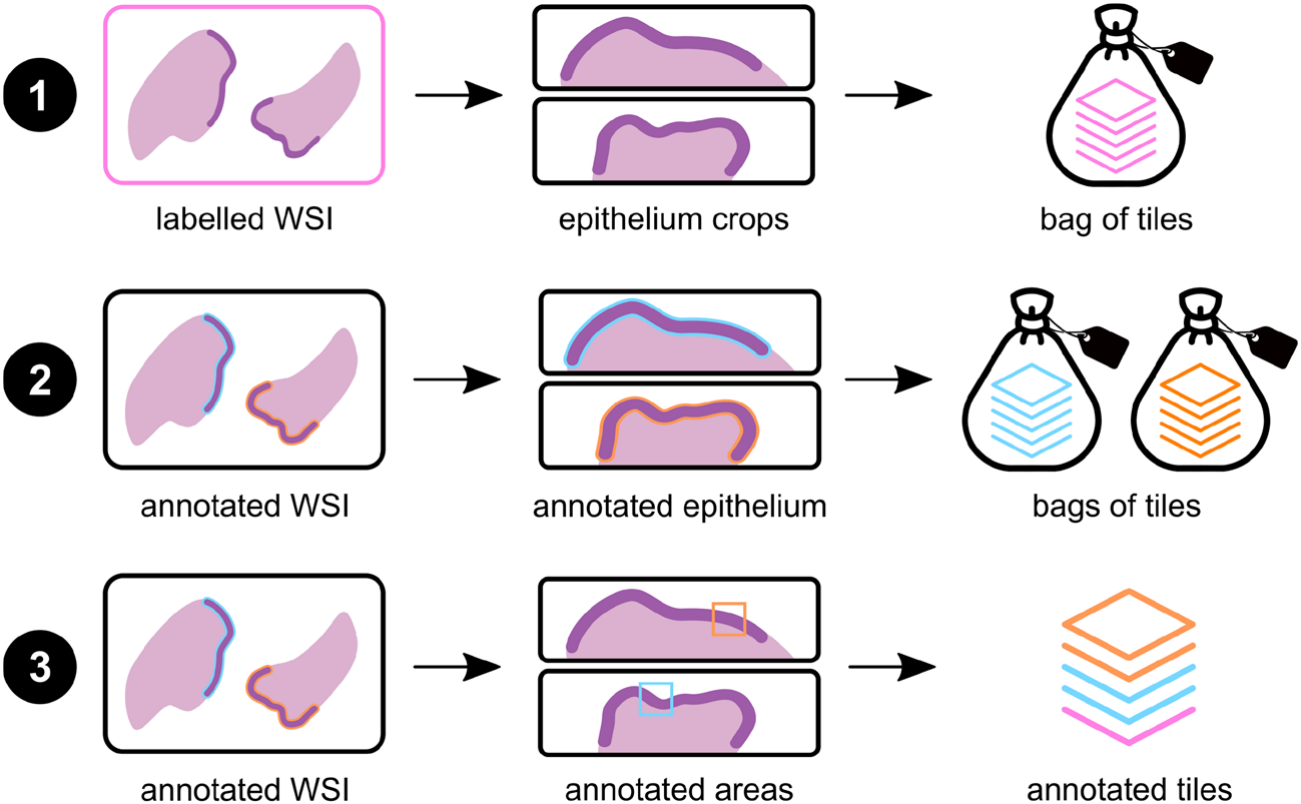
Annotation levels for training: labelled slides (top), annotated epithelium (middle) and annotated tiles (bottom).

In this sense, and following a common approach in computational pathology, we propose a weakly-supervised methodology for cervical dysplasia grading, based on tiles (small areas within the slide) using different levels of training supervision, in an attempt to leverage a big dataset only partially annotated with full details. In this particular case, we define three different levels of data for training, as represented in Fig. 2:

1. Labelled slides (LS): each slide labelled only with the slide diagnosis can be abstracted as a set (or bag) of tiles from the epithelial regions (tiles from non-epithelial regions are not used in the development of the DL model); the labelling of each tile is unknown, but it is assumed that the worst of the unknown tile labels corresponds to the bag labelling (slide diagnosis); Epithelial areas are automatically identified using a (deep) segmentation model;
2. Annotated epithelium (AE): epithelium was delineated and labelled on a subset of the slides; For model development, each labelled epithelial region is considered a set/bag of tiles where the labelling of the set is known but the labelling of the individual tiles is not; The slides with labelled epithelia yield smaller bags than the slides with diagnosis only, which improves the quality of training;
3. Annotated tiles (AT): within the annotated epithelium, smaller regions of interest were delineated, indicating unequivocal tissue areas of non-neoplastic tissue, LSIL and HSIL, from where tiles with known labels were retrieved.

In addition to facilitating labelling, such a scheme can serve two other purposes: acquiring details for training a supervised segmentation model, focusing the assessment of the degree of dysplasia on the regions of interest (ROIs), and adding more information to the tiles to facilitate learning of the classifier.

### Dataset

For this work, we gathered a dataset of 2000 WSIs of LEEP samples and surgical specimens (cervical biopsies were excluded), from 1051 patients. Since the main goal is to grade cervical dysplasia, labelling followed the two-tiered diagnostic system, thus dividing the slides into four classes: non-neoplastic (NNeo), LSIL, HSIL or non-representative (“others”, as the sample example in Fig. 1a).

**Figure 3:**
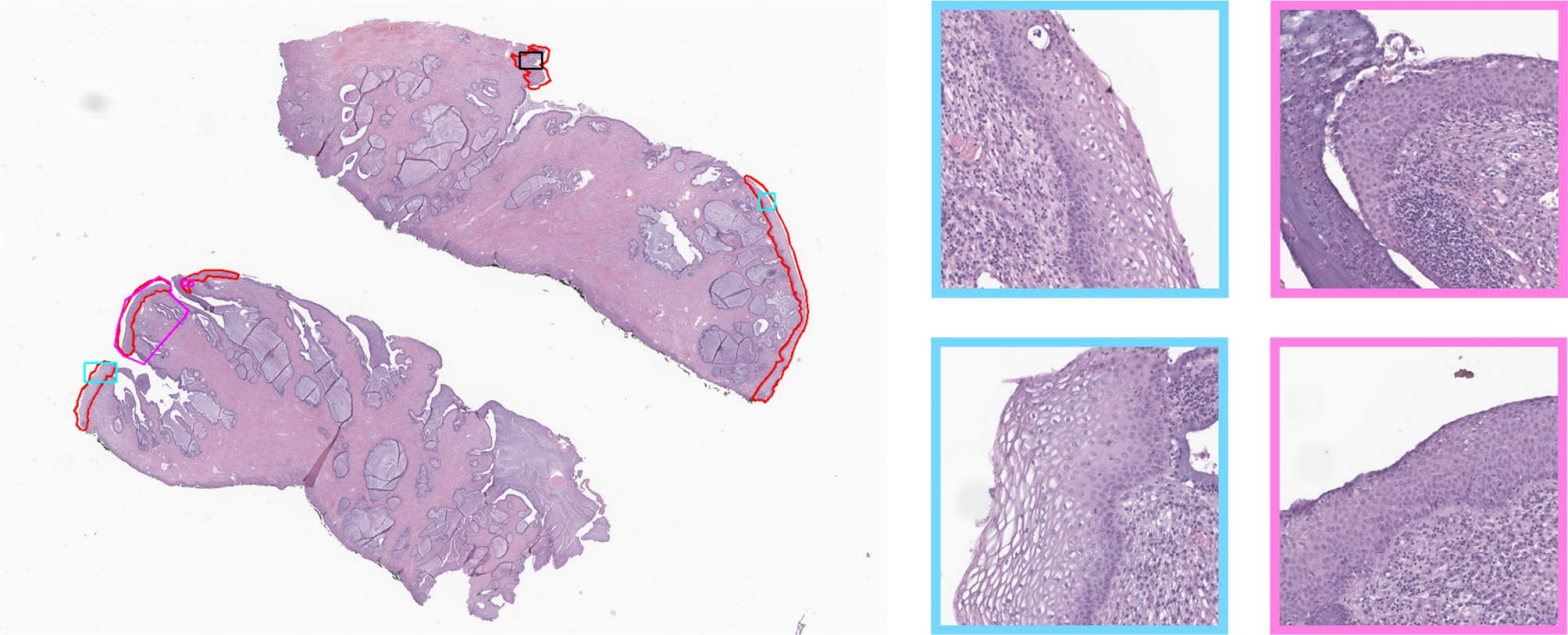
Data annotation example: (red) epithelium areas, (blue) LSIL and respective tiles on the right, (pink) HSIL and respective tiles on the right, (black) uncertain label areas not used for training.

All cases were retrieved from the data archive of the IMP Diagnostics laboratory, Portugal, and were digitised with 2 Leica GT450 WSI scanners, at 40$\times$ magnification (pixel size of $0.26\,\upmu$m$^2$). Diagnostics were made using a medical grade monitor LG 27HJ712C-W and the Aperio eSlide Manager software. The diagnosis was then compared with the original report (which served as a second grader). If both were coincident, no further assessment was performed. In case of difference, or case complexity, the case was rechecked and decided by a third pathologist, specialized in Gynaecopathology. Further, a subset of slides ($n=186$, approximately 10% of the complete dataset) was also manually annotated (like the example in Fig. 3), using the Sedeen Viewer software^35^, delineating epithelium areas and characteristic areas correspondent to the different classification categories. Table 1 summarises the class distribution of annotated and non-annotated data, including the number of WSI, fragment crops (positive fragments, i.e., with epithelium areas, from the NNeo, LSIL and HSIL samples combined, and negative fragments from “others” class), epithelium crops (obtained from annotations or from the segmentation model for the non-annotated set), and the tiles obtained after the pre-processing step described in “Tissue classification”.

To test the entire framework in an independent set of slides, the dataset was divided into a training and a test set, containing 1400 (70%) and 600 (30%) samples, respectively. All the annotated slides were kept to train the models and, from the non-annotated set, all the “others” cases, that do not have epithelium areas to be segmented, were used for testing. In an attempt to address the lack of public datasets of cervical WSI, and to enable direct comparisons of future research, the set of slides used for testing (n = 600) is available upon reasonable request.

**Table 1:** Dataset summary: the number of slides, fragments crops, epithelium areas and tiles per class.

Classes	Slides	Fragment crops	Epithelium areas	Tiles
NNeo	702 (34)	3496 (224)$^{*}$	2082 (52)	35,038 (1413)
LSIL	885 (67)		5620 (240)	58,419 (4868)
HSIL	323 (61)		1885 (91)	17,154 (1087)
Others	90 (24)	184 (88)$^{*}$	–	–
Total	2000 (186)	3680 (312)	9587 (383)	110,611 (7,368)

* The fragment crops are divided into positive samples (that include NNeo, LSIL and HSIL classes) and negative samples (“others” class), if they contain or do not contain epithelium areas, respectively. Epithelium areas were obtained from annotations or from the segmentation model (in case of the non-annotated slides). The number of annotated slides and the corresponding annotations are detailed in parenthesis. Class “others” correspond to non-representative samples, i.e, without epithelial areas and thus, no tiles.

### Learning framework

**Figure 4:**
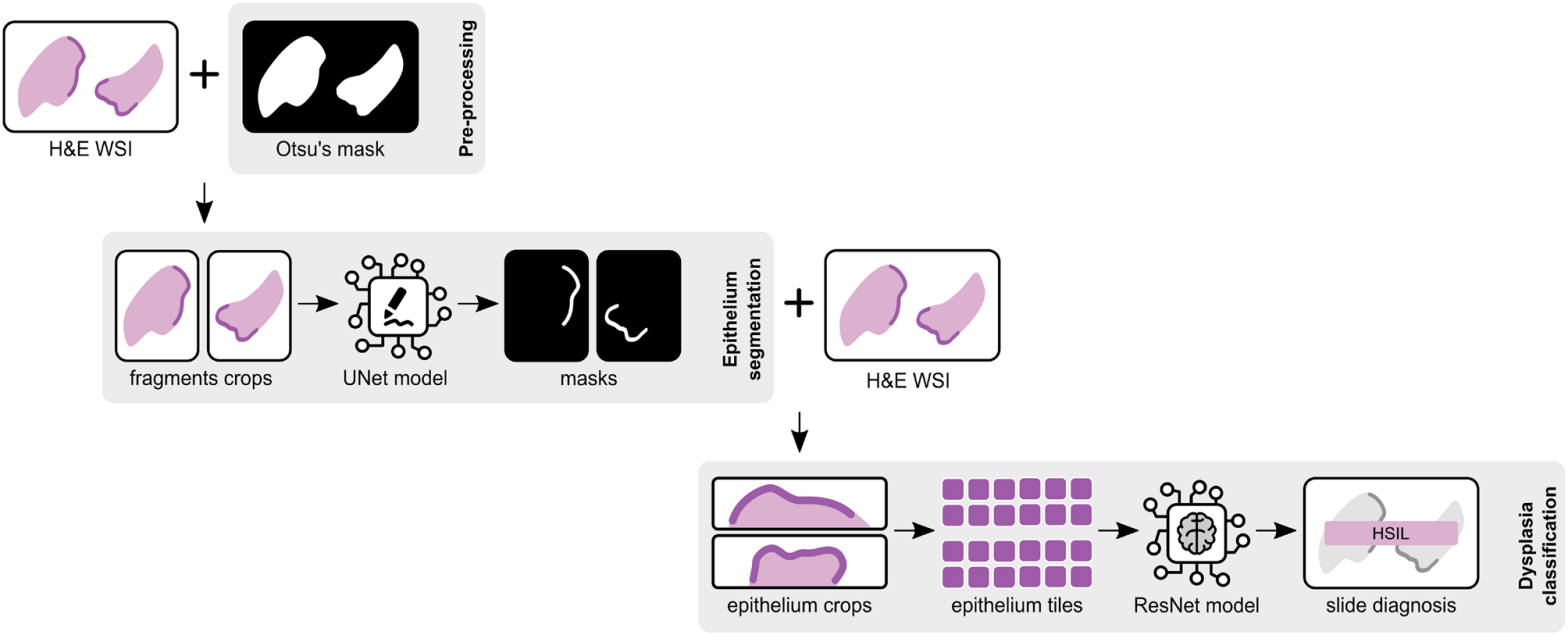
Proposed framework for cervical dysplasia grading on H &E digitised slides. The pipeline includes an epithelium segmentation model, to locate the region of interest within the slide, and a classification model, to grade dysplasia on tiles from the segmented epithelial areas.

The proposed framework for cervical dysplasia grading on H &E digitised slides (Fig. 4) consists of two main parts: an epithelium segmentation model, to locate the region of interest (ROI) to focus on, and a tile classifier, to distinguish normal epithelium, low and high-grade lesions. To analyse a new sample, we first identify the tissue fragments of the slide (Otsu’s preprocessing step) which are then given to the automatic segmentation model to find the epithelium areas to be classified (epithelium segmentation step). With such automatic segmentation step, there is no need for any kind of annotation or manual crop of the epithelium. All these epithelium areas are then converted into tiles that and fed to the classifier, with the final diagnosis for the slide being based on the worst tile, following the multiple instance learning (MIL) assumption (dysplasia classification step). More details of each step of the pipeline are presented below.

#### Epithelium segmentation

**Figure 5:**
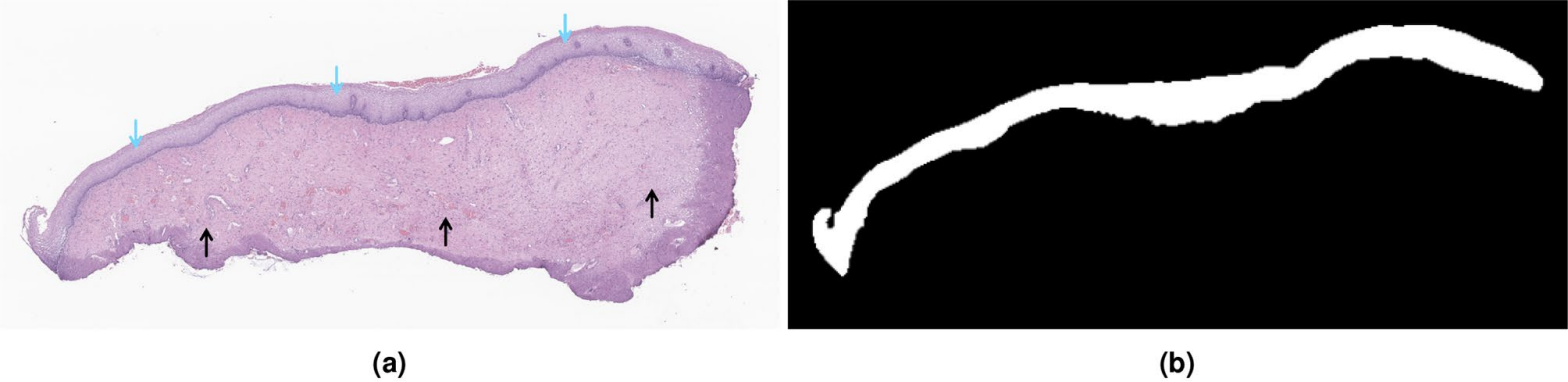
(**a**) Fragment crop, with blue and black arrows indicating the epithelium and stroma areas, respectively; (**b**) epithelium annotation mask.

As mentioned before, relevant features of cervical dysplasia are located in the epithelium, a small portion of the tissue area (marked with blue arrows in the example of Fig. 5a). In this way, assessing the whole tissue area would not only be more time-consuming but would also introduce a lot of noise into the learning process, as the features of the stromal subepithelial areas (black arrows in Fig. 5a) are not directly related to the degree of dysplasia. Thus, epithelium segmentation becomes essential to assess a cervical WSI automatically.

To this end, we propose the U-NET architecture^36^, a standard biomedical image segmentation model^37,38^, trained with fragment crops of the slide, resized to $1024\times 1024$ pixels, to account for the huge variability of slide/tissue/epithelium dimensions between slides. In fact, the amount of tissue in each slide may vary a lot, as well as the ratio of epithelium/submucosa tissue. Moreover, WSIs have huge dimensions (commonly several hundreds of pixels in both width and height) and can not be used in their original size. By using each tissue fragment individually, we can reduce the loss of information of image resize and also the sparsity of images to be segmented. Each fragment is cropped from the slide using an Otsu’s thresholding^39^ mask as a reference to locate tissue and define its limits (Fig. 4). The end goal is to get a mask, such as the annotation one (example in Fig. 5b), to guide the image pre-processing step for the classification model.

For learning the segmentation model, we used the BCE-Dice loss ($L_s$), a standard function used on image segmentation, based on the combination of the binary cross-entropy (BCE) and the Dice loss, defined as:

1 $$\begin{aligned} L_{s} = BCE + Dice\ Loss \end{aligned}$$

where the BCE term is defined as,

2 $$\begin{aligned} BCE\ (y,{\hat{y}}) = - (y \times log({\hat{y}}) + (1-y) \times log(1-{\hat{y}})) \end{aligned}$$

and the Dice loss term is defined as,

3 $$\begin{aligned} Dice\ Loss\ (y, {\hat{y}}) = 1 - \frac{2y{\hat{y}}+\lambda }{y+{\hat{y}}+\lambda } \end{aligned}$$

with $y\in \{0,1\}$ being the target value at each individual pixel, ${\hat{y}}\in \{0,1\}$ the predicted value retrieved by the model and $\lambda$ a smoothing factor (set to 1).

#### Tissue classification

**Figure 6:**
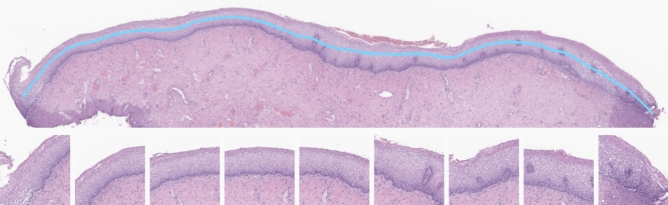
Example of an epithelium crop (top) with some tiles of $512\times 512$ pixels (bottom), sampled along the centre line (in blue) of the epithelium area.

Following the segmentation step, with the output mask (or epithelium annotations in case of annotated samples), we crop smaller regions containing the identified ROIs. However, these images are still too big to be fitted within a GPU and, thus, need to be decomposed, to avoid the loss of key tissue details if the image is resized. Moreover, ideally, the smaller areas to be extracted should include the entire epithelium thickness, so the model can learn the proliferation of basal/parabasal-like cells, as described in “Cervical cancer pathology”. Hence, we use the centre line of the epithelium mask to sample tiles of $512\times 512$ pixels, from the WSI level corresponding to 20$\times$, along the extension of the epithelium (Fig. 6). The centre line of each epithelium is computed by repeatedly eroding and dilate the epithelium mask to refine its skeleton. Then, following the longest path (centre line), we sample points at intervals of 256 pixels on the x-axis, which are then used as the centre of the tiles to be retrieved.

Following a common approach in computational pathology, we propose a semi-supervised learning scheme, based on MIL, using instance bags that can include the tiles of all epithelium areas within a slide, labelled with the slide diagnosis, or tiles of each epithelium area, in the case of annotated epitheliums. Also, taking advantage of some areas annotated explicitly for each class, we included some individually labelled tiles, for full supervision. With such a setup, to train the classification model, we assume that each bag is represented by its worst tile (the tile with the most severe diagnosis), following a generalisation of the MIL assumption. If there is one HSIL tile in the set, then the bag (i.e. the epithelium area or the slide) is diagnosed accordingly. On the other hand, if no tile has dysplasia, the bag is classified as non-neoplastic. It is worth mentioning that for the classification model we only use three classes (NNeo, LSIL and HSIL) since the “others” categories correspond to slides without epithelium areas. To find the most representative tile, the model is firstly used to infer the class of all tiles in the set, which are then ranked. Tile ranking is based on the expected value of the predictions, following the approach of^11,40^. For a tile ${\mathcal {T}}_{s,t}$, where *s* is the index of the set (slide or epithelium) and $t\in \{1,\ldots , t_s\}$ is the tile number within the set, the expected value of the score is defined as:

4 $$\begin{aligned} {\mathbb {E}}({\hat{C}}_{s,t}) = \sum _{i=1}^n i \times p\left( {\hat{C}}_{s,t} = C^{(i)}\right) \end{aligned}$$

where ${\hat{C}}_{s,t}$ is a random variable on the set of possible class labels $\{C^{(1)},\cdots , C^{(n)}\}$ and $p\left( {\hat{C}}_{s,t} = C^{(i)}\right)$ are the *n* output values of the neural network.

To train the classifier, we used the weighted $\kappa$ loss function ($L_c$), based on the quadratic weighted kappa (QWK)^41^, defined as:

5 $$\begin{aligned} L_c =1-\kappa ,\ \ \ \ with\ {\kappa =1-{\frac{\sum _{y,{\hat{y}}=1}^{n}w_{y,{\hat{y}}}\ x_{y,{\hat{y}}}}{\sum _{y,{\hat{y}}=1}^{n}w_{y,{\hat{y}}}\ m_{y,{\hat{y}}}}}} \end{aligned}$$

where *n* is the number of classes, $y\in \{1,2,3\}$ is the actual class, ${\hat{y}}\in \{1,2,3\}$ is the predicted class, $w_{y{\hat{y}}}$ belongs to the weight matrix, $x_{y{\hat{y}}}$ belongs to the observed matrix and $m_{y{\hat{y}}}$ are elements in the expected matrix. The observed matrix is constructed such that $x_{y{\hat{y}}}$ corresponds to the number of samples with grade *y* that are predicted as class ${\hat{y}}$. The expected matrix is calculated as the outer product between the possible actual classes and the predicted ones, normalised so that *x* and *m* matrices have the same sum. The $n\times n$ matrix of weights *w* is computed based on the difference between the actual (*y*) and the predicted (${\hat{y}}$) class, as follows:

6 $$\begin{aligned} w_{y,{\hat{y}}}=\frac{(y - {\hat{y}})^2}{(n - 1)^2} \end{aligned}$$

In this way, class ordinality is taken into account during model training by weighting misclassifications: model predictions closer to the target label are less penalised than more distant ones. In fact, the classification targets are defined upon dysplasia grading, meaning that misclassifying an HSIL as NNeo is worse than identifying it as LSIL.

### Training details

The UNet model was randomly initialised and trained using the adaptive moment estimation (Adam)^42^ optimiser (learning rate of $1\times 10^{-4}$), during 250 epochs, with mini-batches of 4 images, resized for $1024\times 1024$. The classification model was initialised with the ImageNet weights and trained with the Adam optimiser, a learning rate of $1\times 10^{-5}$ and a batch size of 16, for 300 epochs. The models’ performance was evaluated at the end of each epoch to select the best model based on the validation loss and the validation accuracy for the segmentation and classification tasks, respectively. The hyperparameters used during training were empirically set to maximize performance. All experiments were conducted using Pytorch and on a single Nvidia Tesla V100 (32 GB) GPU.

## Results and discussion

### Segmentation model

Considering the lack of literature methods that use the entire slide and the same grading system, to perform a benchmark, we performed several ablation studies to confirm the capabilities of the proposed methodology. In this section, the results of experiments conducted for the segmentation and the classification models are presented individually, as well as the results of the complete framework.

To train the segmentation model we used all of the annotated slides (186), from which we cropped 312 tissue fragments, divided into training and validation sets, in a $\approx 70/30$ ratio respectively, taking into account class (with or without epithelium) and patient stratification (Table 2). All experiments were evaluated in 88 fragments crops, based on 5 metrics at the pixel level: Dice score, intersection over union (IoU), sensitivity, precision and accuracy (Table 3).

**Table 2:** Class distribution of the fragments crops used to train the segmentation model.

Classes	Training set	Validation set	Total
Positives	183 (81.70%)	72 (81.82%)	255 (81.73%)
Negatives	41 (18.30%)	16 (18.18%)	57 (18.27%)
Total	224	88	312

**Table 3:** Performance of the UNet model for epithelium segmentation, trained with different input images (3-channel vs 1-channel) and different loss functions (BCE vs. BCE-Dice Loss).

Model version	Loss function	Dice score (%)	IOU (%)	Sensitivity (%)	Precision (%)
UNet w/ RGB channels	BCE	**74.46**	**60.67**	**75.90**	**74.41**
UNet w/grayscale		70.54	55.63	73.93	68.13
UNet w/saturation channel		53.24	37.47	54.69	55.24
UNet w/ RGB channels	BCE-Dice	**80.64**	**68.17**	82.80	**79.68**
UNet w/grayscale		77.81	64.44	**83.27**	74.73
UNet w/saturation channel		66.86	51.75	72.49	63.45

Bold values indicate best results.

The first experiment used the images’ RGB channels as input, which achieved a Dice score of 80.64%. Next, in an attempt to understand if the difference in the colour of the epithelium could be confusing the model, since some epithelium areas are darker than others, we trained the model with grayscale images, and also using the saturation channel of the HSV colour space. However, as reported in Table 3, the models trained with only one channel did not perform better, so we can conclude that colour information is relevant for the correct distinction between epithelium and submucosa regions, even though the variability mentioned above. Thus, the RGB version was selected as the segmentation model to use for the complete framework. Additionally, we also trained the segmentation model with the standard pixel-wise binary cross-entropy loss (BCE), which showed to be less adequate for the task at hand with any type of input (Table 3).

**Figure 7:**
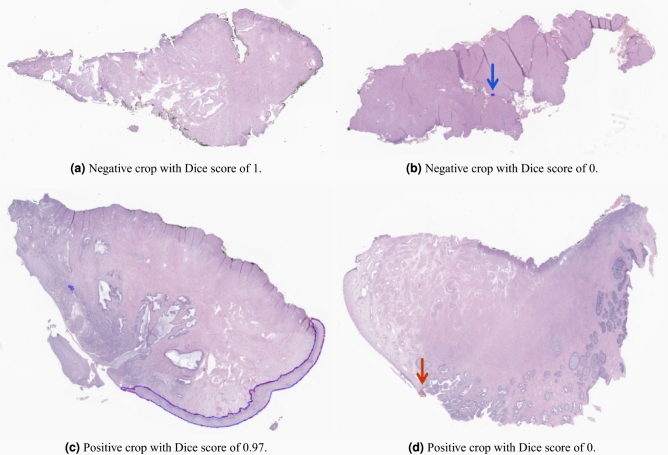
Segmentation model output examples (in blue) in comparison with the ground truth from annotations (in red).

When analysing the predicted masks, as some examples in Fig. 7, it is possible to see that, in most cases, the errors should not significantly affect the next stage of classification, since most errors are a few extra or missing pixels at the edges of the epithelium. Only very rarely does the model fail to recognise a large part of the epithelium or misidentify a significant area.

### Classification model

To train the classification model we used 383 annotated epithelial regions, divided into training and validation sets, also taking into account patient and class stratification, as for the segmentation task (Table 4), resulting in 111 (29%) examples to validate and choose the best classification model. Since we only want to classify epithelium areas, the class “others” is not included.

**Table 4:** Class distribution of the annotated epithelium areas used to train the classification model.

Classes	Training set	Validation set	Total
NNeo	37 (13.60%)	15 (13.51%)	52 (13.58%)
LSIL	177 (65.08%)	63 (56.76%)	240 (62.66%)
HSIL	58 (21.32%)	33 (29.73%)	91 (23.76%)
Total	272	111	383

For this task experiments, and following its promising performance in several previous works on WSI classification^8,11,25,40^, we tested three versions of the ResNet network (with 18, 34 and 50 layers), and two loss functions (the standard cross-entropy, and an ordinal one, the weighted $\kappa$), evaluating the accuracy, quadratic weighted kappa (QWK), sensitivity, precision, F1-score and the mean AUC (Table 5). The best-performing model was the ResNet-34, trained with the weighted $\kappa$ loss function, which achieved an accuracy of 69.64% and a sensitivity of 72.97%.

**Table 5:** Performance of the classification model, trained with annotated epithelium areas as bags/sets of tiles: architectures and loss functions comparison.

Model	Loss function	Accuracy (%)	QWK	Sensitivity (%)	Precision (%)	F1-score (%)	AUC
ResNet-18	CE	67.47	**0**.**56**	68.47	70.86	69.09	0.76
ResNet-34		**67.90**	0.55	**72.07**	**73.23**	**72.50**	**0**.**80**
ResNet-50		66.36	0.50	70.27	71.45	70.66	0.79
ResNet-18	Weighted $\kappa$	69.59	**0**.**58**	72.07	73.21	72.43	0.78
ResNet-34		**69.64**	0.51	**72.97**	**74.86**	**73.65**	**0**.**81**
ResNet-50		67.14	**0**.**58**	71.17	71.47	71.20	0.78

Bold values indicate best results.

In an attempt to leverage the classification learning task, after choosing the best model, we re-trained this version by adding some individual labelled tiles ($n=263$) to the training set, to guide model training with direct supervision of some tiles. In Table 6 (middle row) we can see that, with this addition, all metrics increased, ending with an accuracy of 74.31% and a sensitivity of 74.77%. As expected, by combining the selected tile of each epithelium area, that only has the label of the correspondent bag, with tiles that have a particular labelled associated, the tile selection process was improved.

**Table 6:** Performance of the classification model (ResNet-34) trained with the weighted $\kappa$ loss function and different supervision levels: tiles from annotated epitheliums (AE), annotated tiles (AT) and tiles from labelled slides (LS).

Training data	Accuracy (%)	QWK	Sensitivity (%)	Precision (%)	F1-score (%)	AUC
AE	69.64	0.51	72.97	74.86	73.65	0.81
AE + AT	**74.31**	0.65	74.77	76.44	74.98	0.84
AE + AT + LS	73.78	**0**.**66**	**78.27**	**78.38**	**78.31**	**0**.**85**

Bold values indicate best results.

Lastly, to take advantage of the complete dataset, we re-trained the ResNet-34 also adding bags of tiles ($n=1198$) from the non-annotated slides, using the best epithelium segmentation model (Table 6, last row). It is worth mentioning that, despite adding more data, we also add more noise with the automatic epithelium segmentation and bags of tiles per slide. Nonetheless, the model achieved improved results across all metrics, except the balanced accuracy. In particular, sensitivity had a gain of 3.5%, with the model predicting right more cases from LSIL and HSIL classes combined. When comparing the confusion matrices of both versions (Table 7), we can conclude that the version trained with the non-annotated data (b) misidentified two more LSIL cases and one more normal case. On the other hand, it got seven more LSIL right. However, since HSIL and normal classes are less represented, misclassifications are more penalised with a balanced accuracy.

**Table 7:** Validation set (111 epithelial regions) confusion matrices.

(a) AE+AT				
		Actual class		
		*NNeo*	*LSIL*	*HSIL*
Predicted	* NNeo*	**10**	7	0
	* LSIL*	3	**45**	5
	* HSIL*	2	11	**28**

(b) AE+AT+LS				
		Actual class		
		*NNeo*	*LSIL*	*HSIL*
Predicted	* NNeo*	**9**	5	0
	* LSIL*	4	**52**	7
	* HSIL*	2	6	**26**

Significant values are in [bold].

### Complete framework

Finally, we tested the complete framework with an independent set of slides ($n=600$), using the best epithelium segmentation model (UNet with RGB channels) and the overall best classification model (ResNet-34 trained with the complete dataset). Here, the output of the segmentation model is used, not only for ROI identification but also to classify slides as “others”. Since these samples do not have exocervical epithelium areas, if the segmentation model result is empty, then the slide is automatically classified accordingly.

From Table 8, it is possible to verify that the segmentation model only misses ROIs in two LSIL cases. However, in the non-representative cases, the model has identified 28 cases correctly out of 66, meaning a balanced accuracy of 71.03% for identifying negative and positive samples. From the over-segmented cases, i.e. the cases without exocervical epithelium where the segmentation model identifies some area of interest, the classification model does not recognise any as HSIL and classifies most of them as non-neoplastic. Thus, the overall framework achieves a balanced accuracy of 63.75%, precision of 71.02%, sensitivity 68.67% and an F1-score of 68.18%.

**Table 8:** Test set (600 slides) confusion matrix using the complete framework, with all classes.

		Actual class			
		*NNeo*	*LSIL*	*HSIL*	*“Others”*
Predicted	* NNeo*	**126**	21	0	22
	* LSIL*	73	**202**	24	16
	* HSIL*	5	25	**56**	0
	*“Others”*	0	2	0	**28**

Significant values are in [bold].

When excluding the “others” class, the classification model achieved a balanced accuracy of 71.07%, a QWK of 0.67, precision of 74.15%, sensitivity of 72.18%, F1-score of 72.11% and a mean AUC of 0.85%, being in line with the validation performance reported in “Classification model”. Therefore, we can conclude that the errors of the segmentation model still have some impact on the overall performance of the model, especially on precision and sensitivity. In fact, if the segmentation model misses some relevant area, the classifier would be misled.

## Conclusion and future work

In this work, we propose a weakly-supervised methodology for grading cervical dysplasia (non-neoplastic, LSIL, HSIL and non-representative cases), using different levels of training supervision, in an effort to gather a bigger dataset without the need of having all samples fully annotated. With the first step of segmentation, we can identify ROI to focus on for the classification, allowing the use of non-annotated WSI for training, and the automatic diagnosis of unseen cases. Then, the classifier can diagnose the dysplasia grade from tiles of those areas.

Nonetheless, despite the overall acceptable performance of the complete framework on the test set, further efforts should focus on the improvement of both parts individually, but also on how to better link them. In fact, from the reported results we can conclude that there is some noise being propagated from the segmentation model to the classifier, weakening it. In that sense, an end-to-end training framework can possibly improve the results of the segmentation model by penalising them based on classification quality. Moreover, more information on the heterogeneity of epithelium types within a WSI could be used as an extra layer of weak supervision to guide tile selection.

## Data Availability

The test set (600 samples), from the IMP Diagnostics dataset, used in the current study is available on reasonable request through the following email contact: cadpath.ai@impdiagnostics.com.
